# Identification, Genetic Characterization and Validation of Highly Diverse HIV-1 Viruses for Reference Panel Development

**DOI:** 10.3390/v13071417

**Published:** 2021-07-20

**Authors:** Jiangqin Zhao, Hanxia Huang, Sherwin Lee, Viswanath Ragupathy, Santanu Biswas, Christelle Mbondji-wonje, Xue Wang, Alex Jiang, Indira Hewlett

**Affiliations:** Laboratory of Molecular Virology, Division of Emerging and Transfusion Transmitted Diseases, Center for Biologics Evaluation and Research, Food and Drug Administration, 10903 New Hampshire Ave, Silver Spring, MD 20993, USA; Hanxia.Huang@fda.hhs.gov (H.H.); Sherwin.Lee@fda.hhs.gov (S.L.); Viswanath.Ragupathy@fda.hhs.gov (V.R.); Santanu.Biswas@fda.hhs.gov (S.B.); Christelle.Mbondji@fda.hhs.gov (C.M.-w.); Xue.Wang@fda.hhs.gov (X.W.); Alexjiang8715@gmail.com (A.J.)

**Keywords:** HIV, recombinant, genetic diversity, subtype, phylogenetic analysis, p24, viral load, diagnostics, HIV reference panel

## Abstract

The continued diversification of HIV poses potentially significant challenges to HIV diagnostics and therapeutics. The dynamic evolution of emerging variants is highlighted in countries such as Cameroon in West Central Africa, where all known subtypes and circulating recombinant forms (CRFs) have been shown to be prevalent. We obtained several hundred HIV-positive plasma and viruses from this region for characterization and identification of highly divergent HIV strains. A total of 163 viral strains were cultured to high titers and high volumes using donor peripheral blood mononuclear cells (PBMCs). Initially, 101 viruses representing 59 strains were well characterized and categorized. Results showed that the viral load (VL) range was 0.36–398.9 × 10^7^ copies/mL, p24 values was 0.2–1134 ng/mL. Phylogenetic analysis of thirty-six near full-length HIV-1 genomic sequences demonstrated that most recombinants were highly diverse CRF02 containing unique recombinant forms (URFs). There were seven viral isolates identified as pure subtype/sub-subtypes (F2, A1, G, and D), six as CRFs (CRF06, CRF18, and CRF22), and ten as URFs. These extensively characterized reagents reflect the current dynamic and complex HIV epidemic in Cameroon and provide valuable insights into the potential phylogenetic evolutionary trend of global HIV molecular epidemiology in the future. These materials may be useful for development of HIV validation and reference panels to evaluate the performance of serologic antigen and nucleic acid assays for their ability to detect and quantitate highly divergent HIV strains.

## 1. Introduction

The continued diversification and evolution of HIV poses significant challenges to diagnostics and therapeutics [1,2,3]. Recent studies demonstrated that HIV-1 subtypes and major recombinants should be considered in the evaluation of new HIV therapeutics, prevention modalities, and vaccines [4]. Study of the diversity and evolution of HIV is essential for development of reference material to improve diagnostics and for pandemic preparedness. Cameroon is a country in West Central Africa where HIV-1 infection is pandemic, and the natural reservoir of HIV-1 group M, N, O and P has been identified. The dynamic evolution of emerging variants is demonstrated by the prevalence of all known subtypes, circulating recombinant forms (CRFs), and unique recombinant forms (URFs) in this region. The genetic diversity of HIV-1 appears wide-ranging in Cameroon where all group M clades and CRFs including CRF01_AE(01), CRF02_AG(02), CRF06_cpx(06), CRF09_cpx(09), CRF11_cpx(11), CRF13_cpx(13), CRF22_01A1(22), CRF36_cpx(36) and CRF37_cpx(37) have been identified, and novel URF mosaic structures were also identified [5,6,7,8,9,10,11,12,13,14,15,16]. Among them, CRF02_AG is a predominant strain and represents over 65% of HIV infections with an additional 26% classified as URFs [6,7,8,9,10,11,12,13,14,16]. Co-circulation of different subtypes and CRFs in Cameroon results in the continuing emergence of new recombinants, novel URFs [5,17] and these strains have spread to different geographic regions worldwide [18,19,20].

HIV-1 genetic diversity has been shown to affect diagnostics, blood safety, patient monitoring, therapeutics, and vaccine development [4,21,22,23,24,25]. Our laboratory initiated characterization of HIV-1 in 2002 using a serological survey in Douala and Yaoundé in Cameroon [26], and has collected several hundred HIV-1 positive plasma samples since 2005. A variety of HIV-1 recombinants have been identified from the various studies [11,12,15,27]. In this study, we cultured HIV-1 viruses using peripheral blood mononuclear cells (PBMCs) to generate high-titer and large volumes of viral samples that have been stored as neat culture supernatants. These large-scale viral stocks represent highly divergent HIV-1 materials that reflect the current dynamic and complex HIV-1 epidemic in Cameroon as a basis for the development of comprehensive panels. These materials can be used to develop a variety of HIV reference panels for manufacturers and research labs to evaluate their assays for detection of highly diverse strains for diagnostics and study of virus evolution.

## 2. Materials and Methods

### 2.1. Collection and Cell Culture

Plasma specimens were collected from many clinical sites in Cameroon, including Bamenda (ARC), Buea (BDSH), Limbe (LPH), Limbe (LB), Medical Diagnostic Center (MDC), and a few villages in Cameroon (NYU) in 2005–2017. All specimens were collected with ethical approval of the collection protocol and granted from the local institutional review board. The specimens used for this study had all been previously obtained for another purpose and were de-identified for the analysis. For some specimens, viruses were successfully cultured in freshly PBMCs of buffy coat received from HIV seronegative blood donors from the NIH Blood bank. PBMCs were activated with 10 µg/mL PHA in RPMI medium containing 10% FBS for 3 days. The details of virus preparation followed the UNAIDS protocol [28] and are described in our previous publications [29,30]. Viral cultured supernatants for HIV-1 viruses were generated and collected at different timepoints with a variety of volumes and stored after measuring the p24 antigen or viral load (VL) values. Cell-free viruses with high titers in large volumes were collected, aliquoted in 1.0 mL, and stored at −80 °C. The storage inventory was established based on the type of specimen, storage capacity, tests results, and depletion of usage [31].

### 2.2. Specimens Characterization

#### 2.2.1. Viral Load (VL), p24, and AlphaLISA Assays

All cultured supernatants were initially tested using commercially available FDA-approved nucleic acid and p24 antigen assays. All VL values were determined using an in-house Abbott RealTime HIV-1 assay on the m2000 system (Abbott Molecular, Des Plaines, IL, USA) for automated extraction of viral RNA from supernatant with a detection limit of 50 HIV-1 RNA copies/mL [32]. The HIV-1 p24 Antigen capture assay (PerkinElmer Life Sciences, Boston, MA, USA) was used to measure supernatant virus levels and to monitor HIV appearance in culture supernatants at various times post-infection for virus isolation. Analytical p24 value was determined via the least squares fit to the standard curve at an absorbance equal to the cutoff defined by the manufacturer (i.e., mean negative control O.D. + 0.050, LOD 3.5 pg/mL). The p24 antigen was also evaluated using the AlphaLISA assay [33]. In using each test kit, a serial 10-fold dilution was performed in base matrix HIV negative diluent (SeraCare Inc., Milford, MA, USA) in siliconized Eppendorf tubes prior to testing. All conventional assays were performed in accordance with the manufacturer’s instructions.

#### 2.2.2. RNA Extraction, RT-PCR, and Sequencing

Viral RNA was extracted from an aliquoted supernatant using the QIAamp viral RNA mini kit according to the manufacturer’s protocol (Qiagen Inc., Valencia, CA, USA). Reverse transcription (RT) and nested polymerase chain reactions (PCRs) were performed with the SuperScript III RT-PCR system (Life Technologies, Carlsbad, CA, USA) to generate four overlapping DNA amplicons across the entire HIV genome for near full-length genomic sequences as described previously [15,27]. PCR products were initially treated with ExoSAP-IT reagent (Affymetrix Inc., Santa Clara, CA, USA) prior to applying Sanger sequencing by primer walking using the ABI Prism BigDye Terminator Cycle Sequencing kit and ABI PRISM 310 Genetic Analyzer (Applied Biosystems, Foster City, CA, USA). Alternatively, next-generation sequencing (NGS) was performed using ExoSAP-IT treated PCR amplicons pooled in equimolar amounts from four amplicons. Briefly, the concentration of mixed DNA amplicons was measured by using the Qubit dsDNA BR Assay System (Covaris, Woburn, MA, USA). Two nanograms of DNA product was processed for NGS sample preparation by using an Illumina Nextera XT DNA Sample Preparation Kit and NGS was performed using a MiSeq v2 kit (500 cycles) to produce 2 × 250 paired-end reads (Illumina, San Diego, CA, USA).

#### 2.2.3. Bioinformatics Data Analysis

Near full-length genomic sequences for each virus were generated using the Vector NTI software (v.11.5.2, Life Technologies, Grand Island, NY, USA) and De Novo pipeline in the CLC Bio (v6.0.6, CLC bio, Cambridge, MA, USA) as described previously [15,27]. Consensus contig sequences were generated from short reads using an in-house De Novo assembly pipeline. The consensus sequences were aligned using Vector NTI, and where necessary, manually edited after visual inspection. The sequences were finally confirmed using the combination of consensus contig sequences from Sanger or NGS raw reads. Identified sequences were aligned with HIV-1 reference strains of different subtypes, sub-subtypes and CRFs derived from Los Alamos HIV sequence database (LANL) (A1_AB253429, A1_U51190, A2_AF286237, A2_AF286240, B_K03455, B_U63632, C_AF110967, C_U46016, D_M27323, D_K03454, F1_AF005494, F1_AF077336, F2_AJ249237, F2_AJ249236, G_EU697904, G_AF084936, H_AF005496, H_AF190128, J_AF082394, J_AF082395, K_AJ249235, K_AJ249239, 01_AF197340, 01_AF197341, 02_AJ28613, 02_L39106, 04_AF049337, 04_AF119820, 06_AF064699, 06_AJ288982, 09_AY093607, 09_AY093605, 11_AF492623, 11_AY371149, 18_AF377959, 18_AY586541, 22_JN864049, 22_AY371165, 32_DQ167215, 32_AY535660, 36_EF087994, 36_EF087995, 37_AF377957, 37_EF116594). Various tools (RIP, jpHMM, and HIV Blast) in LANL or NCBI Genotyping with author’s revised references were used to initially analyze near full-length HIV genomic sequences [34,35,36]. Some of the sequences were removed from the reference sequence panel to clarify the phylogenetic tree. The viral nucleotide positions correspond to the reference strain HXB2 (GenBank Accession Number K03455) used for the study and referenced throughout this manuscript. Sequences were aligned in the CLUSTAL W program, and phylogenetic analysis was performed in MEGA 6.1 software using the neighbor-joining method as described previously [37,38].

The newly reported HIV-1 genomic sequences in this publication are available in GenBank under the following accession numbers: MN153475-MN153497, MT349406-MT349419.

## 3. Results

### 3.1. Virus Culture and HIV-1 Storage

The viruses were cultured to get high-titer and high-volume using PBMCs, supernatants were collected multiple times at 7, 14, 21, and 28 d post-infection. The supernatant containing HIV particles was directly tested for p24 value prior to store as neat culture supernatant at −80 °C. A total of 163 different genotypes were cultured in our laboratory covering the years 2005–2017 for different lengths of time; the high titer viruses were aliquoted and stored for research and panel development use. In this study, 101 cultures representing 59 different genotypes were initially characterized according to criteria of genotyping, large volume, different time points post-infection, and collection year.

According to prior genotyping data obtained from plasma specimens, currently cultured viral stocks include four isolates identified as subtypes (B, D, G), five as sub-subtypes (A1 and F2), seven as CRFs (02, 06, 09, 18, 22, and 36), and many as URFs (01+22, 02+09, 02+22; 02+36, G+02, B+22, A1+G+02, B+02, D+02, F2+02, 01+02).

### 3.2. HIV-1 Viruses Represent a High Viral Load and p24 Antigen

The total of 101 viruses (representing 59 strains at different time points post-infection) were quantified for VL and p24. As shown in Table 1, nine strains were harvested at three timepoints of week 1, 2, 3, or 4 post-infection, respectively. Seventeen strains were harvested at timepoints of either week 2 and 3, or week 3 and 4, the rest of the 12 strains were collected at week 3 (post-infection 22–27 days).

Viral load of the nine strains (week 1, 2, and 3) representing 24 viruses were >0.36 × 10^7^ copies/mL (*n* = 23, >Log 6.56 copies/mL). The highest VL was 329.6 × 10^7^ copies/mL (Log 9.52 copies/mL) for 06CMARC10 virus in week 2, and the highest average VL from three timepoints was 211.3 × 10^7^ copies/mL (Log 9.31 copies/mL) for 06CMARC10 strain. Overall, VL range of 101 viruses was 0.36–398.9 × 10^7^ copies/mL (Log 6.56–9.60 copies/mL, average VL 65.2 × 10^7^ copies/mL), whereas 15 viruses are below the LOD, and 10 viruses were not tested. In general, 75% of the viruses had very high viral load values and the peak of VL was observed at week 3. HIV-1 p24 antigen was detected in each specimen and showed a high value from 0.2 to 173.2 ng/mL (Average 31.7 ng/mL). For further evaluation, a nanotechnology based-ELISA assay, AlphaLISA, was used to test p24 in all specimens; the data showed that p24 values ranged from 0.2–1134 ng/mL (Average 108.6 ng/mL).

Good correlation was observed between presence of p24 antigen and VL in cultured viruses at most timepoints. Specificity was 100% with various viruses in all three tests (Table 1). Viral RNA quantitation for VL was used to quantitate viral particles, and p24 antigen testing allowed us to measure HIV-1 p24 antigen in these virus stocks. We are currently performing a collaborative study with multiple labs to quantitate viral RNA in these viral stocks using their different assay systems to more accurately evaluate the HIV-1 VL for value assignment and for downstream panel development.

### 3.3. Phylogenetic Analysis Determines Diverse HIV-1 Viruses

**Figure 1:**
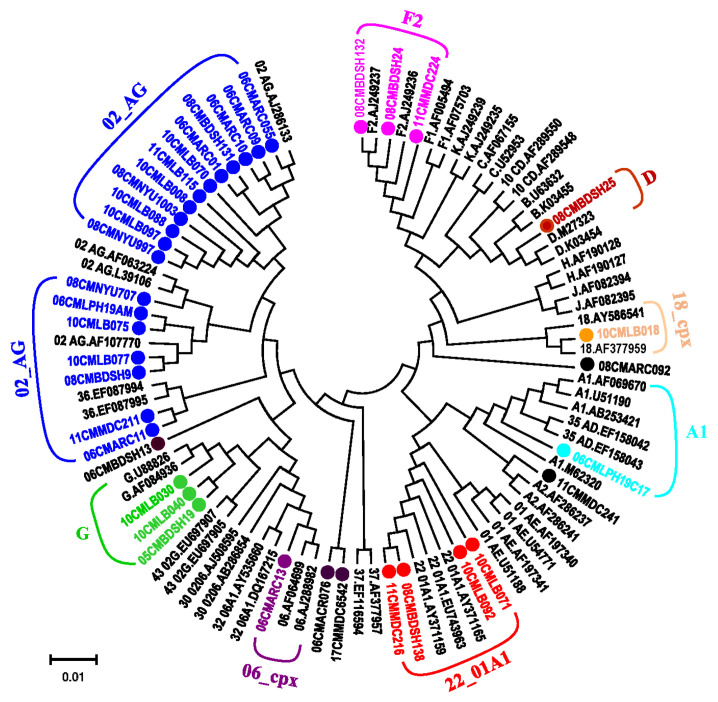
Phylogenetic tree of near full-length HIV-1 genome sequences. Phylogenetic analysis of the near full-length genome sequence was performed using the neighbor-joining module with Kimura’s two-parameter method and bootstrap analysis (1000 replicates); the bootstrap values above 70% are used. The reference sequences of subtypes and CRFs indicated as black were obtained from Los Alamos National Labs (LANL) HIV sequence database (www.hiv.lanl.gov, accessed on 29 April 2021) to construct the tree. Some reference sequences have been omitted for clarity. The new identified isolate sequences are color coded and indicated as dots “•”.

**Figure 2:**
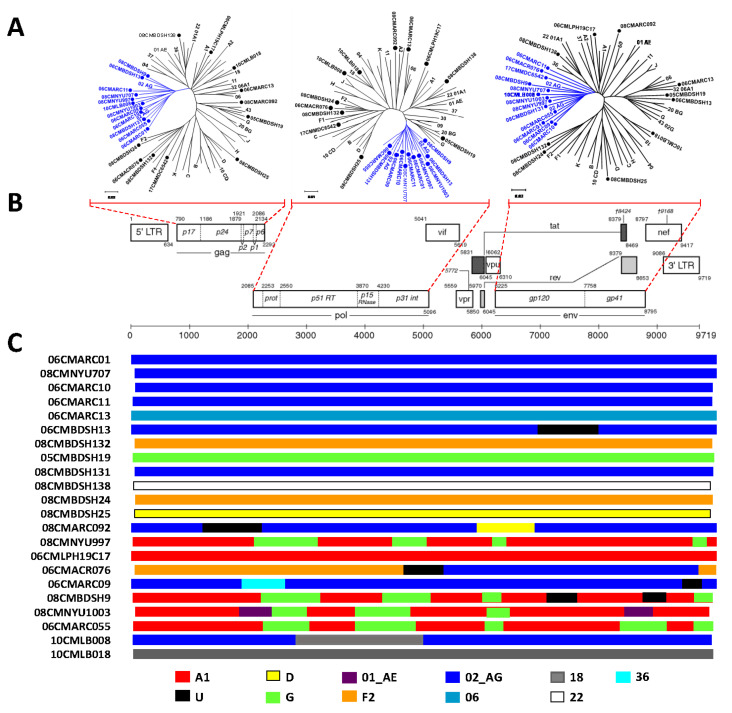
Diagram of the genomic structure of near full-length genome and subtype of gag, pol, and env regions. (**A**) Phylogenetic trees of regions gag, pol, and env regions. The trees correspond to genome segments in gag (HXB2 positions 790–2292), pol (HXB2 positions 2085–5096), and env region (HXB2 positions 6225–8795). References for the subtypes, sub-subtypes, and CRFs are listed in methods and materials and shown as black. The new identified sequences are indicated as dots “•” and cluster of CRF02_AG are shown as blue in the tree. (**B**) Reference structure of the HIV-1 genome HXB2 (K03455). (**C**) Diagram of the mosaic composition. Breakpoint locations are based on the HXB2 number engine. Each of the resulting genome was analyzed by SimPolt, jpHMM, and Genotyping separately, recombinant structures of each strain were determined and colored according to subtype.

In this study, a total of 36 strains were analyzed by near full-length genome sequencing (Figure 1, Table 2). Sequence comparison analysis of those sequences determined 82.1–86.1% of identity to HXB2 genome. Phylogenetic analysis of those sequences confirmed that all sequences clustered to the reference sequence with a high bootstrap value. As shown in Figure 1, nineteen isolates were deeply clustered in the CRF02_AG cluster; the most common recombinants were CRF02 or CRF02 containing URF (CRF02+CRF06, CRF02+F2, CRF02+G). Six of these viruses contain CRF02 clusters. There were seven isolates identified as subtype or sub-subtypes (A1, F2, G, and D), six as other CRFs (CRF06, CRF18, and CRF22), and ten as URFs. Three strains (08CMBDSH24, 11CMMDC224 and 08CMBDSH132) clustered with sub-subtype F2. The viral strains of 05CMBDSH19, 06CMLPH19C17, 08CMBDSH25, 10CMLB018 and 08CMBDSH138 clustered to subtypes or CRFs of G, A1, A2, D, CRF18, and CRF22, respectively. Phylogenetic analysis of each genomic region of gag, pol, and env genes revealed a cluster of CRF02 segments representing complicated genomic composition (Figure 2).

The recombinant breakpoints of near-full length genomes were determined and the mosaic structures shown to be composed of sub-subtypes (A1, D, F2, and G), recombinants (CRF02, CRF06, CRF18, and CRF22) revealing the presence of ten unique recombinant forms (Figure 2C). For example, the most complex mosaic pattern was found for 08CMARC092, being composed of CRF02, D, and U recombination, and 08CMARC076, being composed of F2, U, and CRF02 (Figure S1).

## 4. Discussion

The dynamic evolution of emerging HIV-1 variants in Cameroon could potentially impact the global molecular epidemiology of the HIV epidemic in the future. To study the emerging diversity in Cameroon, we collected several hundred HIV-positive plasma and isolated viruses from PBMC of HIV-positive individuals. A total of 163 viral genotypes were cultured to high-titer and high-volume and were aliquoted and stored for further assay development use. A total of 101 viruses representing 59 strains were analyzed for viral load and p24 antigen and characterized by near full genome sequencing. Test results showed that those viruses represented high viral load (average 65.2 × 10^7^ copies/mL) and p24 values (average 70.15 ng/mL). Thirty-six HIV-1 full-length genomic sequences were identified and characterized; sequence comparison analysis determined those HIV-1 genomic sequences representing 82.1–86.1% of identity to HXB2 genome. Bioinformatics studies further demonstrated that the most common recombinants were CRF02_AG or CRF02 containing URF (CRF02+CRF06, CRF02+F2, CRF02+G) whereas nineteen of these viruses contained CRF02_AG clusters with complicated genomic mosaic composition. There were seven pure subtypes or sub-subtypes (F2, A1, G, and D), nineteen CRFs (CRF02, CRF06, CRF18, and CRF22), and ten URFs. Current studies provide reference materials for highly diverse HIV strains including a variety of subtypes/sub-subtypes/CRFs/URFs that have been extensively characterized. Since the most well-known HIV subtypes and recombinants were reported from Cameroon in West Central Africa, our materials reflect the current HIV dynamic and complex global epidemic; many circulating subtypes, sub-subtypes, and CRFs identified in this study can be included in future new HIV reference panels.

Conventional detection for HIV-1 nucleic acid test methods are based on PCR technology designed to target highly conserved sequences within the HIV-1 genome, the Integrase (INT) region of the Polymerase gene and the Long Terminal Repeat (LTR) region. The first and second WHO International HIV-1 Subtype Reference Panels were developed more than 15 years ago and are currently still in use for validation and calibration of diagnostic reagents [39,40,41]. However, the RNA genome of HIV-1 exhibits a high degree of genetic variability, and panels need to be updated to reflect the predominant strains circulating worldwide. Currently, over 100 CRFs have been identified, and mosaic genomic composition of the new strains have become even more complex [34]. A high-frequency of occurrence of specific mutations can result in under-quantitation or lack of detection by nucleic acid tests and is also suggestive of resistance to HIV-1 antiretroviral therapies. Price et al. reported that the viral loads among persons infected with subtype A is higher than subtype C, and the indicating study of infecting HIV-1 subtype is important for designing new therapeutic and prevention technologies [4]. Therefore, there is a need to update diagnostics and donor testing assays to be able to detect and quantitate the emerging strains. For this purpose, a new generation of reference standards will provide a tool to enhance assay development, standardization, and validation efforts.

## 5. Conclusions

The well-characterized viruses described in the current study will provide reliable materials to be used for development of a variety of HIV-1 reference reagents for HIV donor screening, viral load, and diagnostic assays. These reagents will be helpful to manufacturers of HIV diagnostics and donor screening tests to ensure that they are able to detect and quantitate major CRFs and URFs circulating worldwide. These efforts will further contribute to maintaining blood safety and ensuring effective patient management.

## Figures and Tables

**Table 1:** Detection of viral load (VL), p24, and AlphaLISA.

Patient ID	Year	VL (107 Copies/mL)				p24 (ng/mL)				AlphaLISA (ng/mL)				Assay’s Agreement
		W1	W2	W3	W4	W1	W2	W3	W4	W1	W2	W3	W4	
06CMARC01	2006	1.4	92.4	322.7	-	0.6	49.4	112.3	-	3.9	272.3	0.0 **	-	yes
06CMARC07	2006	0	6.1	5.6	-	2	3.2	1.9	-	23.5	34.7	21.8	-	yes
06CMARC09	2006	147.2	80.8	64.8	-	56.5	91.4	27	-	290.4	330.1	60.4	-	yes *
06CMARC10	2006	120.8	329.6	183.4	-	58.5	110.1	93.6	-	130.3	0.0 **	0.0 **	-	yes
06CMARC11	2006	12.6	27.4	14.7	-	5.9	14.2	6.5	-	0.9	4	1.8	-	yes
06CMARC13	2006	0.4	1.8	1.2	-	0.2	0.9	0.8	-	0.3	1	0.7	-	yes
08CMBDSH24	2008	17.5	121.6	22.5	-	8.2	10.5	13.1	-	29.7	150.8	40.8	-	yes
06CMLPH19C17	2006	34.6	56.7	67.7	-	13	21.4	18.5	-	101.6	59.8	136.2	-	yes *
06CMLPH19AM	2006	N.D.	N.D.	N.D.	-	1.7	63.2	107	-	13.3	128.1	253.6	-	yes
06CMARC071	2006	25	-	65.3	-	8	-	28.2	-	58.3	-	8.4	-	yes *
06CMARC076	2006	25.2	-	111.7	-	9.5	-	41.2	-	57.6	-	1134	-	yes
08CMBDSH131	2008	-	91.7	93.6	-	-	24.1	26.3	-	-	32.8	28.7	-	yes *
05CMBDSH19	2005	-	0.4	2.5	-	-	0.2	0.8	-	-	0.5	3.1	-	yes
08CMNYU707	2008	-	53.6	87.3	-	-	25.6	35.2	-	-	274.7	479.1	-	yes
08CMNYU871	2008	-	100.5	52.8	-	-	25.2	18	-	-	53.2	36.1	-	yes
08CMNYU997	2008	-	1.5	1.8	-	-	0.3	0.5	-	-	1.4	2	-	yes
08CMNYU1003	2008	-	37.3	47.9	-	-	19.6	24.6	-	-	0	70.4	-	yes
10CMLB008	2010	-	-	41.3	14.1	-	-	21.1	9.1	-	-	70.6	10.9	yes
10CMLB022	2010	-	-	100.6	0	-	-	131.1	37.7	-	-	275.1	40.7	yes
10CMLB030	2010	-	-	83.6	85.4	-	-	18.6	23.4	-	-	48.7	56.4	yes
10CMLB031	2010	-	-	180.8	52.8	-	-	107	32.6	-	-	217.5	60.9	yes
10CMLB040	2010	-	-	162.6	48.1	-	-	120.9	6.1	-	-	228.5	0	yes
10CMLB070	2010	-	-	168.4	35.1	-	-	133.4	39.6	-	-	323	140.4	yes
10CMLB075	2010	-	-	106.4	0	-	-	98.9	32.7	-	-	261.4	108.9	yes
10CMLB092	2010	-	-	N.D.	N.D.	-	-	39.8	12.5	-	-	271.4	41.6	yes
10CMLB097	2010	-	-	N.D.	N.D.	-	-	4.2	1.7	-	-	66.1	22.9	yes
10CMLB034	2010	-	-	73.6	-	-	-	27.8	-	-	-	246.9	-	
10CMLB011	2010	-	-	17.3	-	-	-	16.3	-	-	-	414.3	-	
10CMLB012	2010	-	-	41.5	-	-	-	10.5	-	-	-	82.5	-	
10CMLB029	2010	-	-	298.6	-	-	-	173.2	-	-	-	318.8	-	
10CMLB068	2010	-	-	78	-	-	-	92.9	-	-	-	189	-	
10CMNYU488	2010	-	-	163.7	-	-	-	73.2	-	-	-	207.5	-	
08CMARC092	2008	-	-	111.7	-	-	-	27.7	-	-	-	288.4	-	
08CMBDSH132	2008	-	-	51.4	-	-	-	22.8	-	-	-	65.6	-	
08CMBDSH138	2008	-	-	20.3	-	-	-	15.2	-	-	-	68	-	
08CMBDSH139	2008	-	-	83.6	-	-	-	30.6	-	-	-	199.6	-	
08CMBDSH25	2008	-	-	398.9	-	-	-	85.4	-	-	-	580.6	-	
08CMBDSH9	2008	-	-	44.2	-	-	-	23.8	-	-	-	50.9	-	

* 2 of 3 are correlated well; ** Over Limit of Detection (LOD); VL., Viral Load (10^7^ copies/mL); N.D., not done; W, week, “-”, don’t have timepoint for culture.

**Table 2:** Characterized near full-length HIV-1 genomic sequences.

Patient ID	Year	Cultured (Day)	Size (bp)	Identity (%)	Genotype	GB Acc. No
06CMARC01	2006	21	9083	84.2	2	MN153477
08CMNYU707	2008	21	8855	83.2	2	MN153478
06CMARC10	2006	21	8677	82.1	2	MN153479
06CMARC11	2006	22	8865	85.1	2	MN153480
06CMARC13	2006	22	8857	83.6	6	MN153481
06CMBDSH13	2006	14	8933	83.7	02,U	MN153482
08CMBDSH131	2008	21	8862	84	2	MN153486
08CMBDSH132	2008	21	8853	86.1	F2	MN153483
08CMBDSH138	2008	21	8868	83.7	22	MN153487
05CMBDSH19	2005	22	9017	83.3	G	MN153484
08CMBDSH24	2008	22	8855	84.7	F2	MN153485
08CMBDSH25	2008	21	8936	87.6	D	MN153488
08CMARC092	2008	21	8726	85.4	02,D,U	MN153489
08CMNYU997	2008	21	8906	84.3	02,A1	MN153490
06CMLPH19C17	2006	21	8915	84.3	A1	MN153491
06CMARC076	2006	20	8690	84	02,F2,U	MN153492
06CMARC09	2006	21	8777	83.6	02,36,U	MN153493
08CMBDSH9	2008	21	8923	84	2	MN153494
08CMNYU1003	2008	21	8916	84.1	2	MN153495
06CMARC055	2006	8	8893	84.7	02,A1	MN153496
10CMLB008	2010	21	8910	83.1	02,18	MN153497
10CMLB018	2010	21	8950	85	18	MN153475
10CMLB030	2010	21	8929	83.2	G	MT349406
10CMLB040	2010	21	9015	83.3	G,BG	MT349407
10CMLB088	2010	21	8963	84.4	2	MT349408
10CMLB070	2010	21	9103	83.6	2	MT349409
10CMLB071	2010	21	8822	83.5	22	MT349410
10CMLB075	2010	21	8531	84.1	2	MT349411
10CMLB077	2010	21	8886	83.9	2	MT349412
10CMLB092	2010	21	8247	82.6	22	MT349413
10CMLB097	2010	21	8919	84.2	2	MT349414
11CMLB115	2011	28	8868	84.7	2	MT349415
11CMMDC211	2011	24	8888	84.2	22	MT349416
11CMMDC224	2011	24	8854	84.4	F2	MT349417
11CMMDC241	2011	24	8768	83.4	A1,U	MT349418
06CMLPH19AM	2006	21	8913	84.9	2	MT349419

## Data Availability

The data presented in this study are available on request from the corresponding author.

## Supplementary Materials

The following are available online at https://www.mdpi.com/article/10.3390/v13071417/s1, Figure S1: Mosaic structure of recombinant forms.

## Disclaimer

The funder had no role in the initiation, design, performance, analysis, writing, or interpretation of the results of this study, and the decision to submit the manuscript for publication was made solely by the authors. Conflicts that the editors consider relevant to the content of the manuscript have been disclosed. The findings and conclusions in this article have not been formally disseminated by the Food and Drug Administration and should not be construed to represent any Agency determination or policy.
