# Supplementary figures and images for: Identification, Genetic Characterization and Validation of Highly Diverse HIV-1 Viruses for Reference Panel Development
Source: Viruses. 2021 Jul 20;13(7):1417. doi: 10.3390/v13071417 (PMC8310377; doi:10.3390/v13071417)

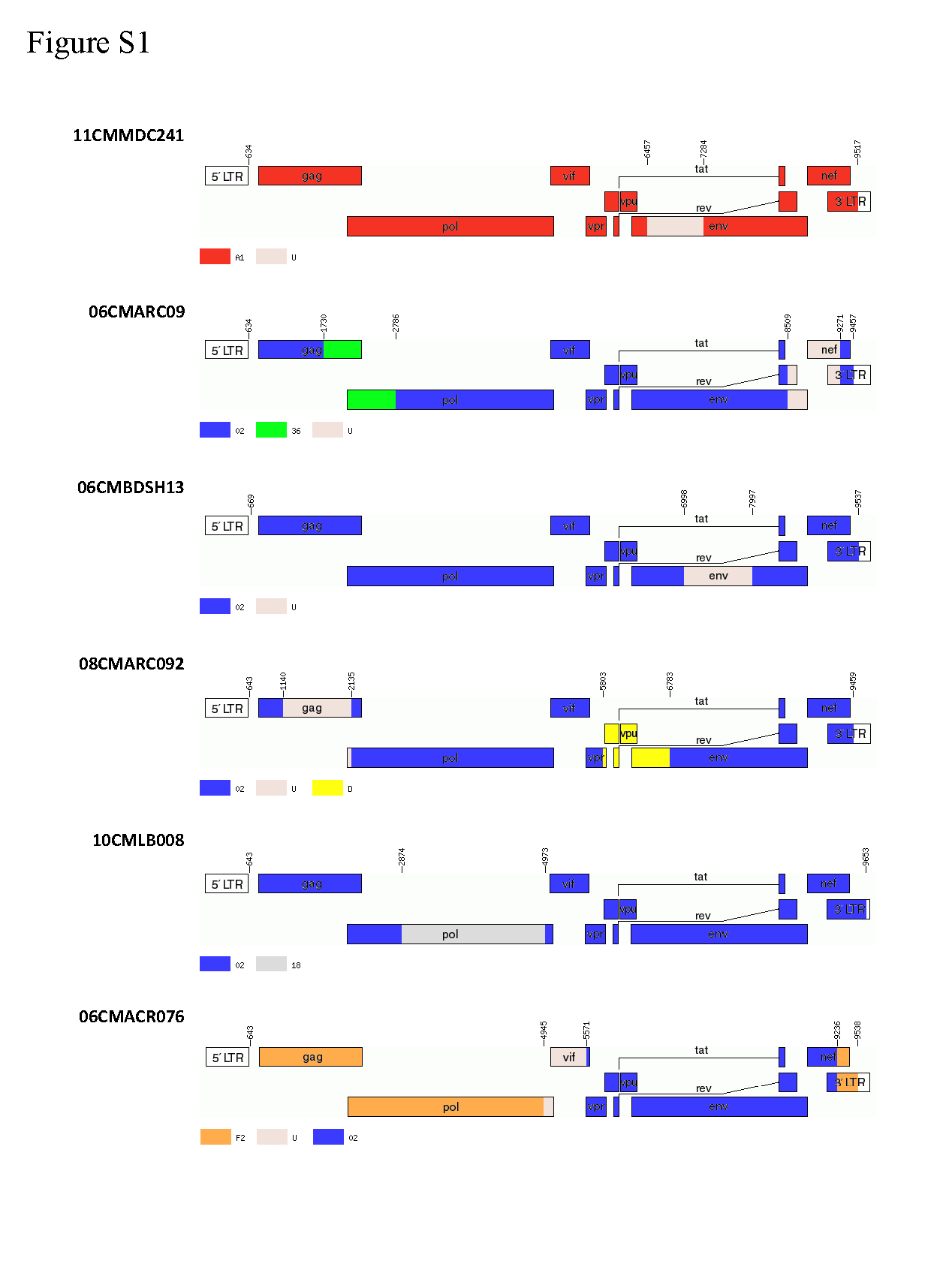

Supplement: Supplementary file 1 [file viruses-13-01417-s001.zip › viruses-1225768-supplementary.tif]
